# circ_0008285 Regulates Glioma Progression via the miR-384/HMGB1 Axis

**DOI:** 10.1155/2023/1680634

**Published:** 2023-08-03

**Authors:** Manli Yan, Caihong Hu, Qi Hu, Heran Ma, Changjiang Lei, Yamei Liu

**Affiliations:** ^1^Department of Internal Medicine, The Fifth Hospital of Wuhan, Wuhan 430050, China; ^2^Department of Internal Medicine, Wuhan Hospital of China University of Geoscience, Wuhan 430074, China; ^3^Department of Surgery, The Fifth Hospital of Wuhan, Wuhan, Hubei 430050, China; ^4^Qilu Cell Therapy Technology Co., Ltd., Jinan 250100, China; ^5^Department of Oncology, The Fifth Hospital of Wuhan, Wuhan 430050, China; ^6^National Research Center of Engineering and Technology for Veterinary Biologicals/Institute of Veterinary Immunology and Engineering, Jiangsu Academy of Agricultural Sciences, Nanjing 210014, China; ^7^Jiangsu Co-Innovation Center for Prevention and Control of Important Animal, Infectious Diseases and Zoonoses, Yangzhou 225009, China; ^8^GuoTai (Taizhou) Center of Technology Innovation for Veterinary Biologicals, Taizhou 225321, China

## Abstract

**Background:**

Recent studies indicate that circular RNAs (circRNAs) have been implicated in the initiation or progression of a wide spectrum of diseases. In the current study, we explored the potential engagement of circ_0008285 in glioma and investigated the downstream regulators.

**Methods:**

The detection of circ_0008285 level in glioma specimens and cell lines was conducted by quantitative real-time polymerase chain reaction. The chi-squared test was employed to evaluate the relationship between the circ_0008285 level and the clinical features of glioma patients. The roles of circ_0008285 on the proliferation and apoptosis of glioma cells were studied by knockdown experiment. Meanwhile, the regulatory relationship of circ_0008285, miR-384, and high mobility group protein B1 (HMGB1) was explored in glioma cells, and we explored the effects of circ_0008285/miR-384/HMGB1 pathway on glioma cells.

**Results:**

In glioma specimens and cell lines, the expression of circ_0008285 was significantly increased, and a high circ_0008285 level was associated with a larger tumor size and more advanced grading in glioma patients. Furthermore, downregulating circ_0008285 suppressed proliferation and triggered apoptosis of glioma cells, which was associated with a cell cycle arrest at the G1/G0 phase. Mechanism studies indicated that circ_0008285 regulated HMGB1 by sponging miR-384. Functional experiments demonstrated that circ_0008285 promoted the malignant phenotype of glioma cells by miR-384/HMGB1 axis.

**Conclusion:**

Our study revealed circ_0008285 as a novel oncogenic factor in glioma through modulating the miR-384/HMGB1 pathway, suggesting that targeting circ_0008285 could serve as a strategy for glioma management.

## 1. Introduction

Glioma is the most prevalent brain tumor originating from glial cells, which account for approximately 40% of primary brain tumors [1, 2]. So far, surgical removal is a mainstay therapeutic approach for glioma, which has been proven to increase survival [3]. Post-surgical management mainly relies on radiotherapy and pharmacological treatment [2]. Although optimal surgery plus chemo-radiotherapy could help prolong the survival of glioma patients, the high invasiveness of glioma cells is usually accompanied by local tissue invasion, which makes the complete eradication of glioma cells impossible [4, 5]. Cancer recurrence and developed drug resistance are common causes of glioma relapse and treatment failure. The overall survival time of glioma patients remains at a median of 15 months after undergoing standard treatment [2], and the five-year survival rate is as low as 14.6% [6]. For the most aggressive form of glioma, patients with glioblastoma suffer from a dismal prognosis with an estimated 10-year survival rate of 0.71% [7]. However, the detailed molecular mechanisms underlying the aggressiveness and cancer recurrence of glioma remain to be fully studied.

Non-coding RNAs (ncRNAs) have emerged as an intensive research focus in cancer biology. Data indicate that ncRNAs are implicated in many kinds of biological activities, including the occurrence and progression of cancers [8]. Circular RNAs (circRNAs) are a novel category of endogenous ncRNAs featured by a covalently looped structure [9, 10]. Mechanistically, many circRNAs could adsorb microRNA as molecular sponges and in turn, regulate the downstream target genes of microRNAs [11]. Recently, circRNAs have been shown to play critical roles in glioma progression. For example, circPTN overexpression promotes proliferation and stemness in glioma [12]. In contrast, circ-FBXW7 was found to be downregulated in glioblastoma samples [13]. Isoliquiritigenin downregulates circ0030018 to suppress the tumorigenesis of glioma via the miR-1236/HER2 signaling pathway [14]. Besides, circNEIL3 promotes the progression of glioma by mediating the immunosuppressive polarization of macrophage [15]. Unveiling the regulatory mechanism of circRNAs in the progression of glioma could shed light on the potential intervention targets for treatment.

Circ_0008285 is a recently identified circRNA derived from the chromodomain y-like protein locus, which has been reported to be overexpressed in hepatocellular carcinoma and cervical cancer [16, 17]. However, currently, there is no report regarding the expression pattern and the role of circ_0008285 in glioma. We analyzed the GSE146463 database containing glioma specimens and normal tissues and found the significant upregulation of circ_0008285 in glioma samples. Since the role of circ_0008285 in glioma remains unclear, this study aims to investigate the functional engagement of circ_0008285 in glioma cells and the relevant molecular mechanisms. We showed that silencing circ_0008285 impaired the proliferation and survival of glioma cells. Circinteractome prediction indicated an interaction between circ_0008285 and miR-384, which was confirmed by luciferase assay and their expression pattern in tumor samples. We further demonstrated that miR-384 acted as a negative regulator of high mobility group protein 1 (HMGB1). Since HMGB1 is a well-established oncogene promoting tumorigenesis in many cancers [18], our data support the notion that circ_0008285 overexpression enhances the HMGB1 protein level through sponging miR-384 to support the malignancy of glioma cells.

## 2. Methods

### 2.1. Geo Database Analysis

The GSE146463 dataset was downloaded from the GEO database (https://www.ncbi.nlm.nih.gov/gds) and employed to analyze the circRNA expression profile between glioblastoma cells (GC, *n* = 8) and neural progenitor cells (NPC, *n* = 3). The circ_0008285 expression data of NPC and GC cells were analyzed by student's *t*-test.

### 2.2. Clinical Tissue Samples

Tumor specimens were collected during resection surgeries from 64 pathologically confirmed glioma patients who had not undergone radiotherapy and chemotherapy in the Fifth Hospital of Wuhan. Control normal brain tissues were collected from 64 patients with traumatic brain injury during emergency surgeries. All tissues were immediately snap-frozen in liquid nitrogen and then stored at −80°C until further processing. The clinicopathological parameters of the glioma patients and their association with circ_0008285 expression were presented in Table 1. Our study was approved by the ethics committee of the Fifth Hospital of Wuhan. All the patients provided signed informed consent.

### 2.3. Cell Lines and Cell Culture

Human glioma cells (including A172, U251, U87, and SHG44) and human normal astrocytes (NHAs) were acquired from the Cell Bank of the Chinese Academy of Sciences (Shanghai, China). The cells were cultured in Dulbecco's Modified Eagle Medium (Gibco, USA) supplemented with 10% fetal bovine serum (Gibco, USA), 100 *μ*g/mL streptomycin, and 100 U/mL penicillin (Sigma, Germany) under 5% CO_2_ at 37°C. The cells were sub-cultured every three days by trypsinization, and all the experiments were performed using cells within 15 passages. Cell transfection was performed using AAT Bioquest 2000 transfection reagent (Xian, China). Small interference RNAs (siRNAs) were synthesized by Beixin Bioecth (Suzhou, China): si-circ_0008285#1: 5′-ACGGGAAAGGTTGAAAGGATT-3′; si-circ_0008285#2: 5′-AACGGGAAAGGTTGAAAGGAT-3′; si-circ_0008285#3: 5′-GGGAAAGGTTGAAAGGATTGT-3′; and negative control (si-NC): 5′-TTCTCCGAACGTGTCACGT-3′.

### 2.4. Quantitative Real-Time Polymerase Chain Reaction

Gene expression in glioma cell lines (A172, U251, U87, and SHG44) was detected via quantitative real-time polymerase chain reaction (qRT-PCR) using NHAs as normal control cells. Briefly, total RNA was isolated by Trizol reagent (Life Technologies, USA), and cDNA synthesis was performed using a cDNA reverse transcription kit (Takara, Japan). qPCR was performed by SYBR Premix Ex Taq™ kit (Takara) on the QuantStudio qPCR platform (Bio-Rad, USA). The fold change of the target gene was normalized to glyceraldehyde-3-phosphate dehydrogenase (GAPDH) or U6 based on the 2^−*ΔΔ*Ct^ method. The sequences of primers are as follows: circ_0008285 forward: 5′-AGCCGGTCGGAGCTTTATTG-3′, circ_0008285 reverse: 5′-TCCTTTCAACCTTTCCCGTTAAC-3′;

GAPDH forward: 5′-GGAGCGAGATCCCTCCAAAAT-3′, GAPDH reverse: 5′-GGCTGTTGTCATACTTCTCATGG-3′;

miR-384 forward: 5′-AGCGCAGATTCCTAGAAATTG-3′; miR-384 reverse: 5′-CTCAACTGGTGTCGTGGA-3′.

U6 forward: 5′-CTCGCTTCGGCAGCACA-3′; U6 reverse: 5′-AACGCTTCACGAATTTGCGT-3′.

### 2.5. Cell Proliferation Assays

Cell Counting Kit-8 (CCK-8) reagent (Dojindo, Japan) was used to examine the proliferation of U251 and U87 cells. 2 × 10^3^ U251 and U87 cells were inoculated in each well of a 96-well plate. Si-circ_0008285, and/or miR-384 inhibitors were transfected into U251 and U87 cells using AAT Bioquest 2000 transfection reagent (Xian, China) for 48 hours, and the cells were then incubated for different time points. CCK-8 reagent (10 *μ*L) was added at indicated time point, and the cells were further incubated for 2 hours. A spectrophotometer (BioTek, USA) was used to detect the OD value at 450 nm.

For colony formation assay, U251 and U87 cells were plated into 6-well plates at 1500 cells/well and cultured for 14 days. Subsequently, U251 and U87 cells were fixed using 4% paraformaldehyde and stained with 0.25% crystal violet (Sigma). Cells were photographed under a light microscope at 1000 magnification, and the number of colonies (each colony with at least 50 cells) was counted.

### 2.6. Flow Cytometry Analysis of Apoptosis and Cell Cycle

A flow cytometry approach was applied to determine cell apoptosis and cell cycle. An annexin V-FITC/propidium iodide cell apoptosis detection kit (BD Biosciences, USA) was used to detect cell apoptosis. U251 and U87 cells were trypsinized and re-suspended in 500 *μ*L binding buffer, then 1 *μ*L annexin V-FITC and 1 *μ*L propidium iodide were added to the cells for 25 minutes of incubation. Cells were washed once with the binding buffer and then analyzed on the Accuri Cytometer (BD Biosciences). The cell cycle assay kit (Beyotime, China) was used to assess the cell cycle. U251 and U87 cells were fixed with 75% anhydrous ethanol overnight in the fridge. After RNase digestion, the samples were stained using propidium iodide for 30 minutes in the dark and then analyzed on the Accuri Cytometer (BD Biosciences).

### 2.7. Luciferase Assay

The binding sites between circ_0008285 and miR-384, and miR-384 and HMGB1 were tested using the luciferase assay. To construct luciferase reporter vectors, the circ_0008285 and the HMGB1 mRNA 3′ UTR sites complementary to miR-384 were cloned into a Dual-Luciferase vector (pmirGLO, Addgene, USA). AAT Bioquest 2000 transfection reagent (Xian, China) was applied for cell transfection. 48 hours after transfection, the dual-luciferase assay kit (Zeye Biotech, China) was used for the determination of luciferase activity. The proportion of Firefly luciferase activity relative to Renilla luciferase activity was computed.

### 2.8. Western Blot

Protein samples were extracted using cold Radioimmunoprecipitation Assay buffer (Beyotime), and protein quantification was conducted with a BCA kit (Invitrogen, USA). 20 *μ*g of protein sample was separated using Sodium dodecyl-sulfate polyacrylamide gel electrophoresis gel and then transferred to a polyvinylidene difluoride (PVDF) membrane (Millipore, USA). After blocking in 5% non-fat milk for 2 hours, the PVDF membrane was incubated with antibodies against HMGB1 (ab18256, Abcam) and GAPDH (ab9485, Abcam) for 18 hours at 4°C. After washing with phosphate-buffered saline with Tween 20, the membrane was further labeled with a secondary antibody (ab6721, Abcam) for 60 minutes. Protein signals were visualized with an enhanced chemiluminescence detection system (ECL, Pierce).

### 2.9. RNA Pull-Down Experiments

Cells lysates of glioma cells were harvested by RNA Immunoprecipitation (RIP) lysis buffer (Beyotime) and were incubated with 100 nM biotinylated circ_0008285, miR-384, or control oligo for 4 hours. Then the cell lysates were mixed with 80 *μ*L of streptavidin magnetic beads (Sigma) and incubated at 4°C for another 4 hours. The beads were precipitated and washed three times using RIP lysis buffer. The associated nucleic acids on the beads were extracted with Trizol reagent and analyzed by qRT-PCR.

### 2.10. Statistical Analysis

Data are expressed as mean ± SD. A two-tailed unpaired *t*-test was applied to determine the differences between the two groups. One-way analysis of variance was used to determine differences among three or more groups. Chi-squared test was conducted to analyze the association of circRNA level and clinical features in glioma patients. Univariate and multivariate analyses were performed to identify independent variables associated with overall survival. All statistical analyses were performed using the Statistical Package for the Social Sciences version 23.0. *p* < 0.05 was considered statistically significant.

## 3. Results

### 3.1. Elevated circ_0008285 Expression in Glioma Tumor Specimen and Cell Lines

**Figure 1:**
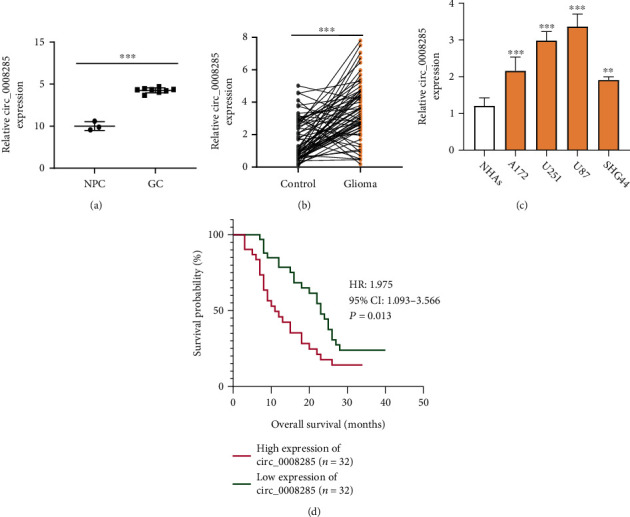
The level of circ_0008285 in glioma specimens and cell lines is significantly increased. (a) By analyzing GSE146463 database, we found that the level of circ_0008285 in glioblastoma cells (GC, *n* = 8) was higher than that in NPCs (*n* = 3). (b) qRT-PCR revealed that the level of circ_0008285 in glioma tumor samples (*n* = 64) was higher than that in normal brain tissues (*n* = 64). (c) Compared with NHAs, the expression of circ_0008285 in glioma cells (A172, U251, U87, and SHG44) was increased significantly. (d) KM-plotter showed that the high expression of circ_0008285 was associated with a poor prognosis in glioma patients. ∗∗*p* < 0.01. ∗∗∗*p* < 0.001.

We initially analyzed the GSE146463 dataset and found that the level of circ_0008285 in glioblastoma cells (GC, *n* = 8) was higher than that in NPCs (*n* = 3; Figure 1(a)). We also collected 64 pairs of glioma samples and non-cancerous normal brain tissues, and qRT-PCR analysis confirmed the overexpression of circ_0008285 in glioma samples (Figure 1(b)). Meanwhile, the elevated circ_0008285 expression was also detected in glioma cell lines (A172, U251, U87, and SHG44) when compared with the normal control cells NHAs (Figure 1(c)). To analyze the correlation of circ_0008285 level and patient survival, 64 glioma patients were assigned into high- and low-expression groups using the median value of circ_0008285 expression as the cutoff. Kaplan–Meier (KM) curve analysis demonstrated that patients with a high expression of circ_0008285 showed a poorer prognosis (Figure 1(d)). Together, these data imply that circ_0008285 overexpression may contribute to the malignant progression of glioma.

### 3.2. circ_0008285 Overexpression Is Associated with Clinicopathological Characteristics in Glioma Patients

To analyze the relationship between circ_0008285 expression level and clinicopathological parameters in glioma patients, glioma patients were divided into high- and low-expression groups based on the median value of circ_0008285 expression (*n* = 32 in each group). Chi-squared analysis revealed the associations between the expression of circ_0008285 and the clinicopathological features (Table 1). The data indicated that the expression level of circ_0008285 was positively associated with tumor size and World Health Organization (WHO) grade, whereas no significant correlation was observed with age and gender. Furthermore, univariate and multivariate Cox models were analyzed to independent risk factors for glioma survival. Despite tumor size (≥3), WHO grading (III–IV), and circ_000828 (high expression) were identified as variables influencing glioma survival in univariate analysis, only WHO grading (III–IV) was identified as the independent risk factor for overall survival of glioma (Table 2).

### 3.3. Down-Regulating circ_0008285 Impairs Proliferation and Survival of Glioma Cells and Induces G1/G0 Arrest

**Figure 2:**
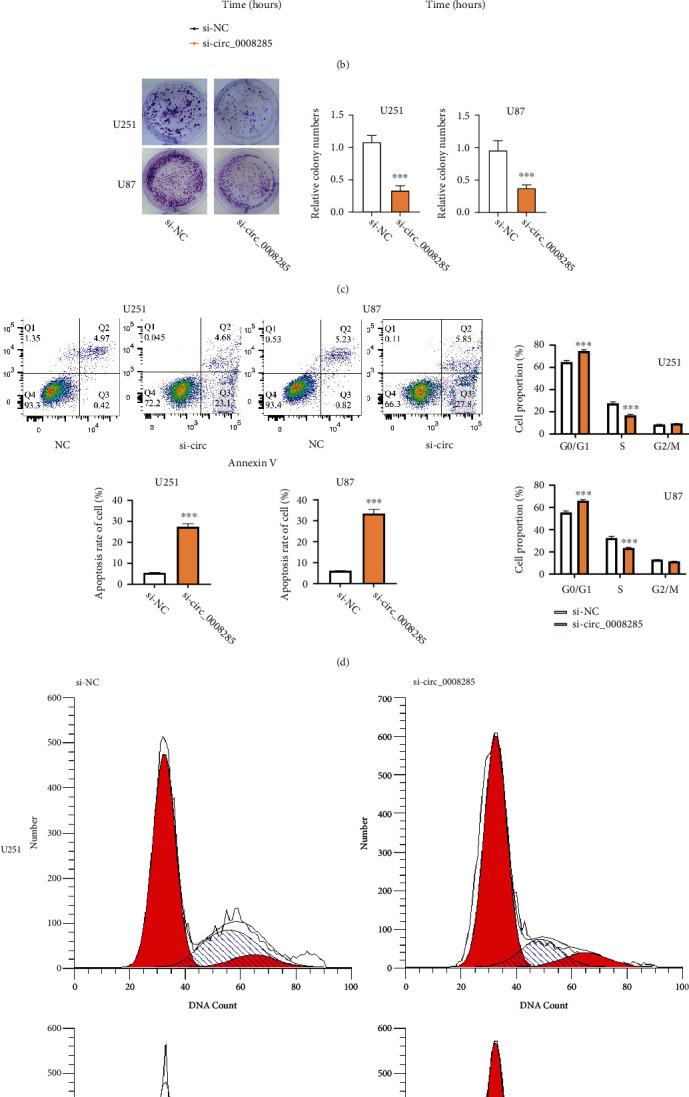
The knock-out of circ_0008285 inhibits cell proliferation, induces apoptosis, and arrests cell cycle in glioma cells. (a) Three siRNAs of circ_0008285 were transfected to glioma cells. Compared with si-circ_0008285#2 and si-circ_0008285#3 and si-circ_0008285#1 showed more profound silencing effect. (b) CCK8 experiment revealed that circ_0008285 silencing significantly inhibited cell proliferation in U251 and U87 cells. (c) Silencing circ_0008285 decreased the formation of cell clones in U251 and U87 cells. (d) Silencing circ_0008285 significantly increased the percentage of apoptosis events in U251 and U87 cells. (e) Silencing circ_0008285 increased the G0/G1 cell percentage and decreased the S cell percentage in U251 and U87 cells. ∗∗*p* < 0.01. ∗∗∗*p* < 0.001.

We designed three siRNAs targeting circ_0008285 (si-circ_0008285#1, si-circ_0008285#2, and si-circ_0008285#3) as well as the si-NC to perform loss-of-function experiment of circ_0008285 in U251 and U87 cells. Using qRT-PCR analysis, si-circ_0008285#1 showed the strongest silencing effect and was chosen for the following experiments (Figure 2(a)). U251 and U87 cell growth was examined through the CCK-8 assay upon circ_0008285 silencing. Compared with the si-NC group, the proliferation of the si-circ_0008285 group was significantly attenuated at different time points (Figure 2(b)). Meanwhile, we performed the colony formation assay to show that si-circ_0008285 transfection suppressed the cologenesis in glioma cell lines (Figure 2(c)). Flow cytometry analysis showed that silencing circ_0008285 promoted apoptotic cell death in both U251 and U87 cell lines (Figure 2(d)). Meanwhile, the percentage of cells at the G0/G1 phase in the si-circ_0008285 group was significantly increased, and the percentage of cells at the S phase was significantly decreased upon silencing circ_0008285 (Figure 2(e)). These findings suggest that the downregulation of circ_0008285 suppresses proliferation and induces apoptosis in glioma cells, and arrests glioma cells at G0/G1 phase.

### 3.4. circ_0008285 Regulates HMGB1 by Sponging miR-384

**Figure 3:**
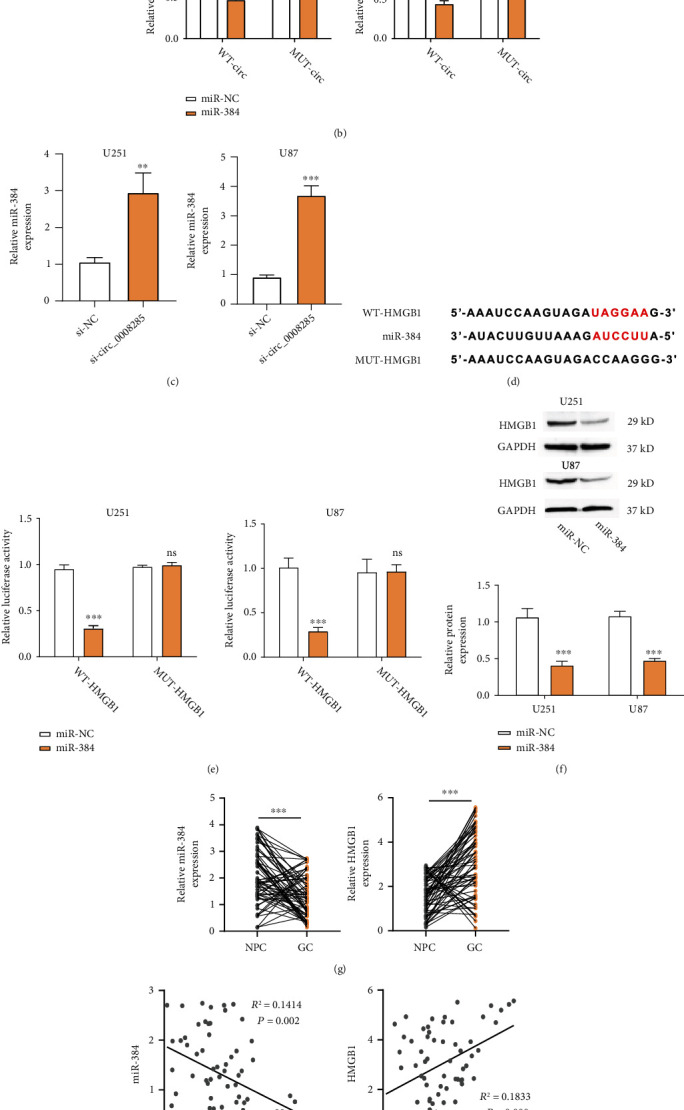
circ_0008285 regulates the expression of HMGB1 protein by targeting miR-384. (a) Circinteractome database prediction of the binding sequence of circ_0008285 and miR-384. (b) Dual luciferase assay using WT-Circ or MUT-Circ vector in the presence or absence of miR384 mimic. (c) miR-384 expression level after circ_0008285 silencing was detected by qRT-PCR. (d) StarBase prediction of miR-384 complementary site at HMGB1 mRNA 3′ UTR. (e) Dual luciferase assay using WT-HMGB1 or MUT-HMGB1 vector in the presence or absence of miR384 mimic. (f) Western blot analysis of HMGB1 expression after miR-384 overexpression. (g) qRT-PCR analysis of HMGB1 and miR-384 in glioma tumor samples was higher than in normal brain tissues. (h) Correlation analysis of circ_0008285, miR-384, and HMGB1 in glioma tumor specimens. ∗∗∗*p* < 0.001.

To search for the targets of circ_0008285, we analyzed the Circinteractome database and identified multiple potential interacting partners of circ_0008285 (Figure S1A), with miR-384 showing the highest prediction score. To narrow down the predicted target, we performed an RNA pull-down assay using biotin-labeled circ_0008285. qRT-PCR assay showed that miR-384 was heavily enriched among circ_0008285 targets in U251 cells (Figure S1B). The predicted interacting sequence between circ_0008285 and miR-384 was shown in Figure 3(a). Luciferase assay demonstrated that miR-384 overexpression repressed the activity in the WT-circ vector, but no effect was observed for the MUT-circ vector with mutated binding sequence (Figure 3(b)), indicating the physical interaction of miR-384 and circ_0008285. Silencing circ_0008285 could increase the miR-384 level in U251 and U87 cells (Figure 3(c)). However, miR-384 overexpression did not affect circ_0008285 expression (Figure S1C), suggesting that miR-384 is a downstream target of circ_0008285.

Through StarBase database analysis, we found several mRNA targets of miR-384 (Figure S2A). RNA pull-down analysis using a biotin-labeled miR384 probe showed the strongest enrichment of HMGB1 mRNA (Figure S2B). The predicted interacting sequence between HMGB1 mRNA and miR-384 was shown in Figure 3(d), and the luciferase reporter experiment confirmed their functional interaction, as miR-384 mimics could inhibit the luciferase activity in the WT-HMGB1 group (Figure 3(e)). The protein level of HMGB1 was also decreased upon miR-384 overexpression in U251 and U87 cells (Figure 3(f)). miR-384 tended to be downregulated, whereas HMGB1 showed upregulation in glioma tumor samples (Figure 3(g)). In addition, HMGB1 mRNA level showed a positive correlation with circ_0008285, whereas miR-384 expression was negatively correlated with circ_0008285 in glioma tumor specimens. Overall, the above results indicated that circ_0008285 could regulate HMGB1 by sponging miR-384.

### 3.5. circ_0008285 Contributes to the Malignant Phenotype of Glioma Cells by Targeting miR-384/HMGB1 Pathway

**Figure 4:**
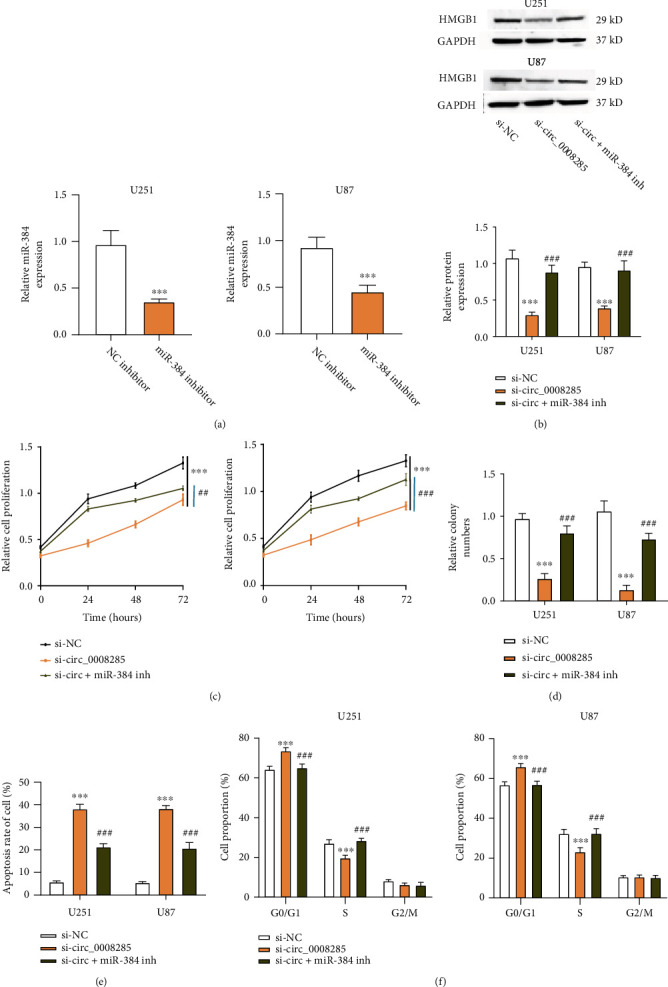
circ_0008285 promotes the malignant phenotype of glioma cells through miR-384/HMGB1 axis. (a) qRT-PCR showed the downregulation of miR-384 upon the transfection of miR-384 inhibitor. (b–f) U251 and U87 cells were transfected with si-NC, si-circ_0008285, and si-circ_0008285 + miR-384 inhibitor. (b) Western blot analysis of HMGB1 protein level in each experimental group. (c) CCK-8 experiment of cell proliferation capacity in each experimental group. (d) Colony formation assay in above experimental groups. (e) Flow cytometry analysis of apoptosis in above experimental groups. (f) Flow cytometry analysis of cell cycle distribution in above experimental groups. ∗∗*p* < 0.01. ∗∗∗*p* < 0.001 versus si-NC. ^##^*p* < 0.01. ^###^*p* < 0.001 versus si-circ_0008285.

Next, we explored whether miR-384/HMGB1axis mediates the role of circ_0008285 in glioma cells. We applied miR-384 inhibitor which could dampen the level of miR-384 in U251 and U87 cells (Figure 4(a)). Then cells were transfected with si-circ_0008285 in the presence or absence of miR-384 inhibitor. Western blot showed a decrease of HMGB1 level upon circ_0008285 silencing, and the level of HMGB1 protein was partially restored in the presence of miR-384 inhibitor (Figure 4(b)), suggesting that circ_0008285 regulates HMGB1 by targeting miR-384 in U251 and U87 cells. CCK-8 assay demonstrated that the inhibitory effect of circ_0008285 silencing on cell growth was attenuated after the application of miR-384 inhibitor (Figure 4(c)). A similar effect was observed using colony formation assay (Figure 4(d)), indicating that circ_0008285 silencing inhibited cell proliferation through modulating the miR-384/HMGB1 pathway. Compared with the siRNA control, si-circ_0008285 increased cellular apoptosis and the percentage of cells at the G0/G1 phase. The administration of miR-384 inhibitor largely abrogated the effect of si-circ_0008285 (Figures 4(e) and 4(f)), indicating that circ_0008285 regulates cell apoptosis and cell cycle progression through miR-384/HMGB1 axis. Collectively, our data suggest circ_0008285 contributes to the malignant phenotype of glioma cells by modulating the miR-384/HMGB1 pathway.

## 4. Discussion

In the current study, we found that circ_0008285 was highly expressed in glioma tissues and was associated with a poor prognosis in glioma patients. Functional experiments suggested that circ_0008285 contributes to glioma cell proliferation and survival by modulating miR-384/HMGB1 pathway and supporting the malignant phenotype of glioma cells.

Recently, increasing studies have reported the functional engagement of circRNAs in different cancers, including glioma [19, 20]. For example, downregulation of hsa_circ_0001836 induces cell death in glioma cells [21], and circRNA PIP5K1A promotes the progression of glioma [22]. In our study, we showed the upregulation of circ_0008285 is indispensable for sustaining the proliferation and survival of glioma cells, which is consistent with the previous data of circ_0008285 in other cancers [16, 17]. Besides, our data revealed the oncogenic role of circ_0008285 in glioma. The knock-out of circ_0008285 inhibited cell proliferation, induced apoptosis of glioma cells, and caused a cell cycle arrest at G1/G0 phase. Thus both previous studies and our data support the notion that the overexpression of circ_0008285 sustains the malignant progression of different cancers.

Mechanism studies showed that circRNAs exert regulatory functions mainly through the miRNA-mRNA axis [23]. For instance, circHECTD1 contributes to glioma progression by regulating the miR-296-3p/SLC10A7 axis [24], and circ-TOP2A regulates glioma progression via miR-346/SUSD2 [25]. In the present study, we performed bioinformatics prediction and confirmed that circ_0008285 serves as a molecular sponge for miR-384 in glioma. Previous studies have shown that miR-384 play some key role in glioma progression [26–28], and it also exerts regulatory roles in other cancers, such as lung cancer [29], prostate cancer [30], and gastric cancer [31]. Our data added novel evidence that miR-384 is a tumor suppressor in glioma, which is suppressed by circ_0008285. However, in other types of cancers, the regulation of miR-384 is mediated by other ncRNAs, indicating that different ncRNA modules are implicated in cancers with different tissue origins.

We further demonstrated that miR-384 could negatively target HMGB1 to regulate the malignancy of glioma cells. Although there were multiple candidate binding partners of miR-384, HMGB1 showed the strongest enrichment with miR-384 and their interaction was validated by luciferase reporter assay. Both circ_0008285 knockdown and miR-384 overexpression could decrease HMGB1 expression. More importantly, HMGB1 expression showed a positive correlation with circ_0008285 and a negative correlation to miR-384 in glioma specimens. HMGB1 is recognized as a damage-associated molecular pattern and expresses highly in autoimmune diseases, tissue injury, and infection [32]. Previous studies have indicated that HMGB1 is implicated in regulating the progression of glioma [33–36]. However, a report claimed that HMGB1 expression does not correlate with the prognosis in glioma [37]. Our study showed that a high level of circ_0008285 expression promotes the level of HMGB1 protein. Since high circ_0008285 expression was associated with poor prognosis in glioma patients, and there was a positive correlation between circ_0008285 level and HMGB1 expression in glioma patients, our data support the notion that HMGB1 overexpression could contribute to the malignant phenotype of glioma cells.

However, several open questions remain to be answered or validated. First, how circ_0008285 becomes deregulated in glioma cells needs to be clarified to manipulate its expression. Whether and how HMGB1 is implicated in the malignant progression of glioma tumors need to be validated using a larger cohort of glioma patient samples and animal models. Furthermore, the oncogenic role of circ_0008285 also needs to be confirmed in a mouse model.

In summary, this study revealed a novel role of the highly expressed circ_0008285 in glioma. Our results suggested that circ_0008285 overexpression sustains glioma cell proliferation and survival by acting as a sponge for miR-384 and maintaining the HMGB1 level. With further validation of this regulatory module in animal models, targeting circ_0008285 could be a potential intervention strategy in glioma.

## Figures and Tables

**Table 1:** The association between circ_0008285 expression level and clinicopathological parameters in glioma patients.

Patients	High circ_0008285 (*n* = 32)	Low circ_0008285 (*n* = 32)	*p*-Value
Age (years)			0.614
≥50	19	17	
<50	13	15	
Gender			0.794
Female	20	21	
Male	12	11	
Tumor size			0.027∗
≥3	23	14	
<3	9	18	
WHO grading			0.009∗∗
I–II	6	16	
III–IV	26	16	

∗*P* < 0.05 and ∗∗*P* < 0.01.

**Table 2:** Univariate and multivariate Cox analyses of independent risk factors for glioma.

Parameters	Univariate Cox analysis			Multivariate Cox analysis		
	Hazard ratio	95% confidence interval	*p*-Value	Hazard ratio	95% confidence interval	*p*-Value
Age (≥50)	1.162	0.758–2.018	0.327			
Gender (female)	1.196	0.648–2.645	0.562			
Tumor size (≥3)	1.526	0.817–2.115	0.039	1.526	0.817–3.115	0.719
WHO grading (III–IV)	2.731	1.275–3.637	0.016	2.078	1.163–3.683	0.011
circ_000828 (high)	1.018	0.669–1.634	0.032	1.416	0.607–2.862	0.367

## Data Availability

Data supporting this research article are available from the corresponding author or the first author on reasonable request.

## Supplementary Materials

Supplementary Materials Full scan of uncropped western blot images.

Supplementary Materials Figure S1. Screening of potential interacting miRNAs of circ_0008285. A. Circinteractome database prediction results of the candidate binding miRNAs of circ_0008285. B. RNA-pull down analysis of the interacting miRNAs using biotin-circ_0008285 probe or control oligo in U251 cells. Data were normalized to the input sample. C. qRT-PCR analysis of circ_0008285 expression level after miR-384 overexpression. ∗∗stands for *p* < 0.01, ∗∗∗stands for *p* < 0.001.

Supplementary Materials Figure S2. Screening of potential target mRNAs of miR-384. A. StarBase prediction results of miR-384 target mRNAs. B. RNA-pull down analysis of the interacting mRNA targets using biotin-miR-384 probe or control oligo in U251 cells. Data were normalized to the input sample. ∗∗stands for *p* < 0.01, ∗∗∗stands for *p* < 0.001.
