# Supplementary figures and images for: circ_0008285 Regulates Glioma Progression via the miR-384/HMGB1 Axis
Source: Int J Genomics. 2023 Aug 3;2023:1680634. doi: 10.1155/2023/1680634 (PMC10415084; doi:10.1155/2023/1680634)

Fig3F


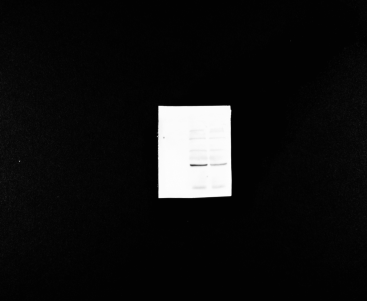

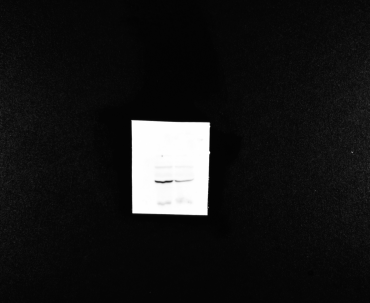


29 kD

HMGB1


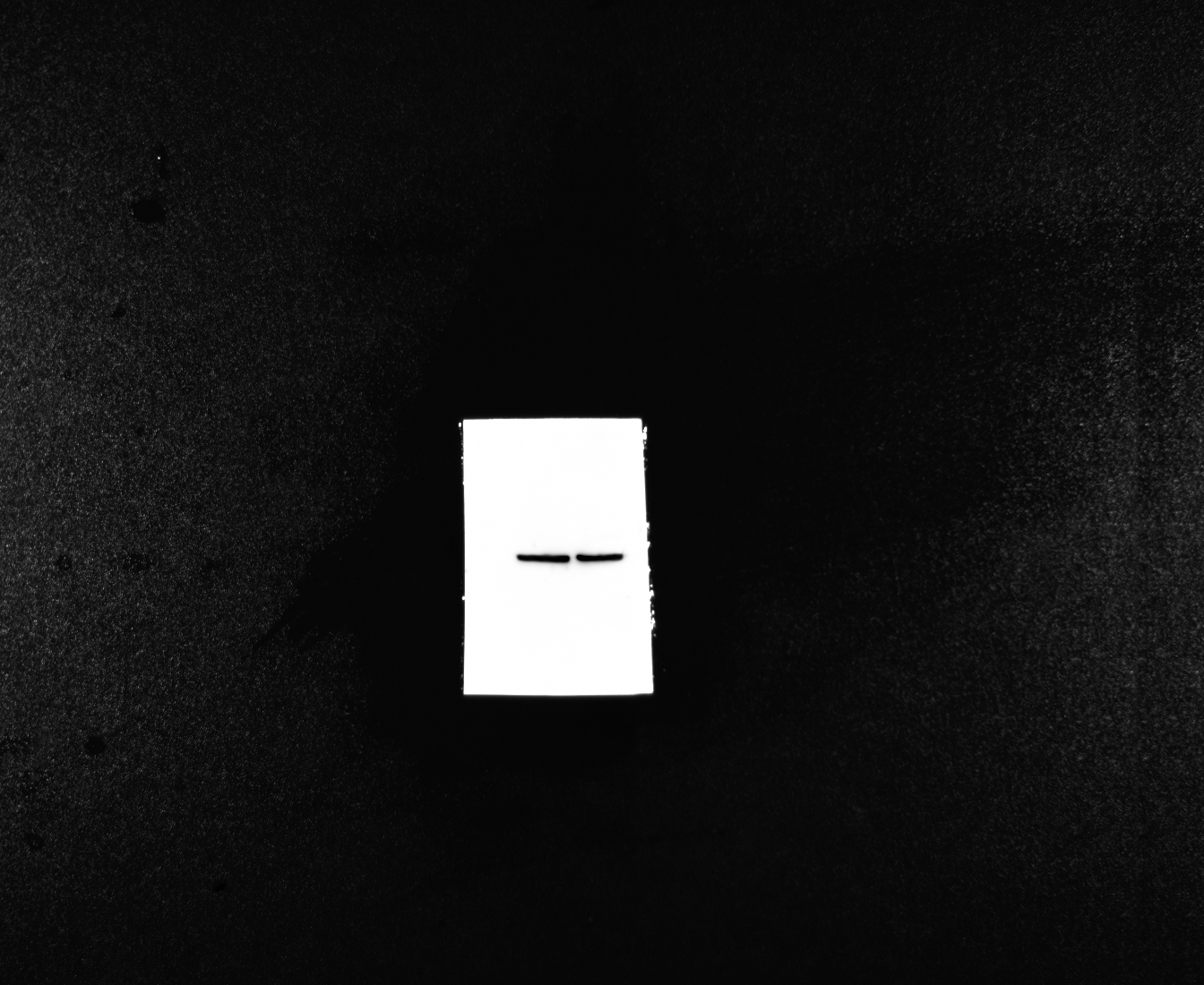

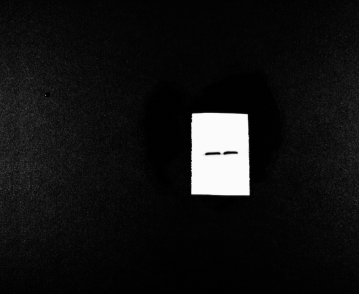


37 kD

GAPDH

Fig4C


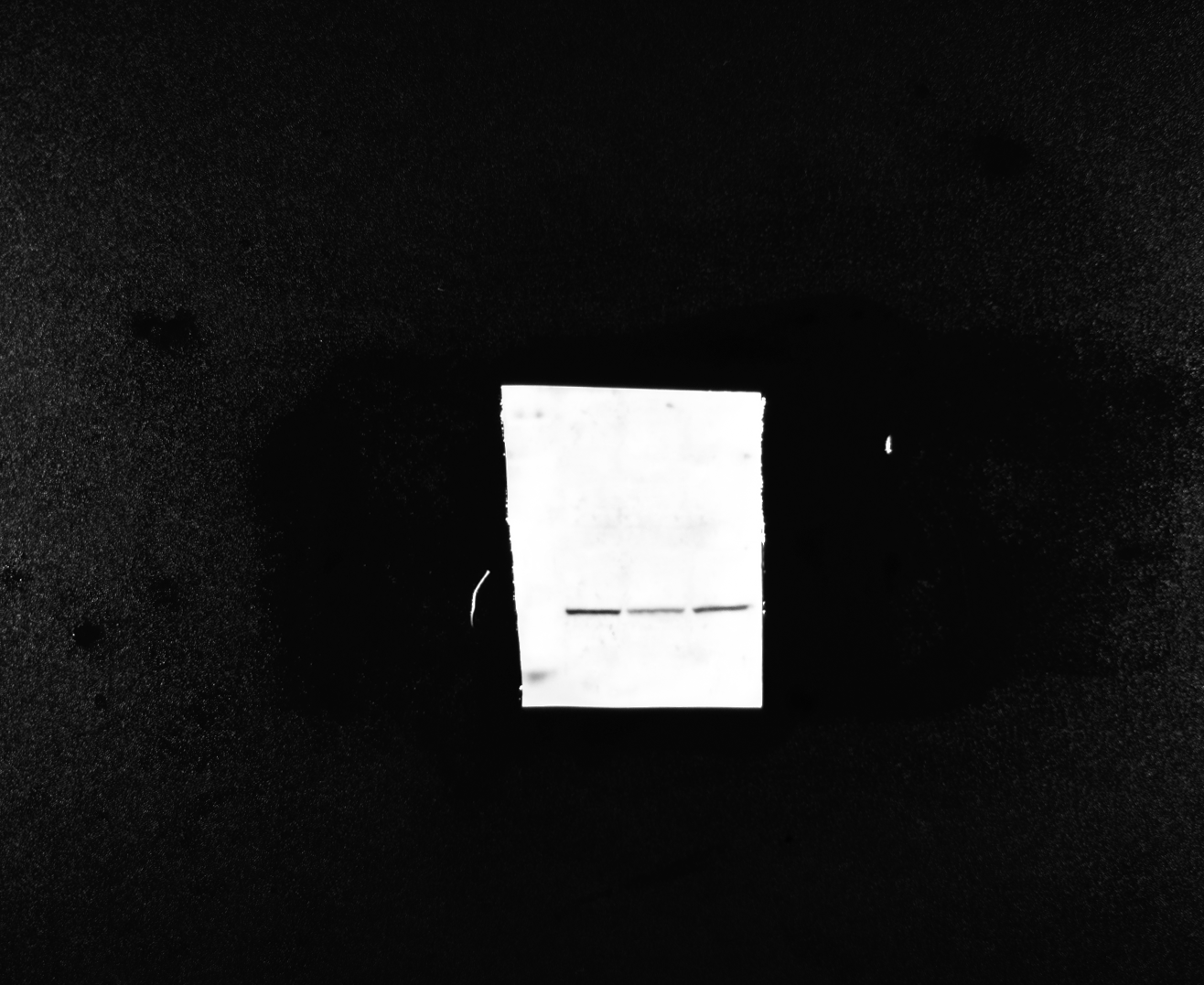

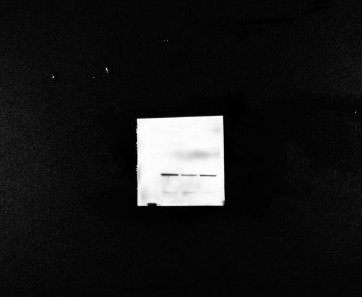


29 kD

HMGB1


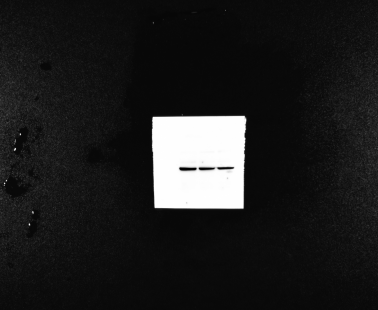

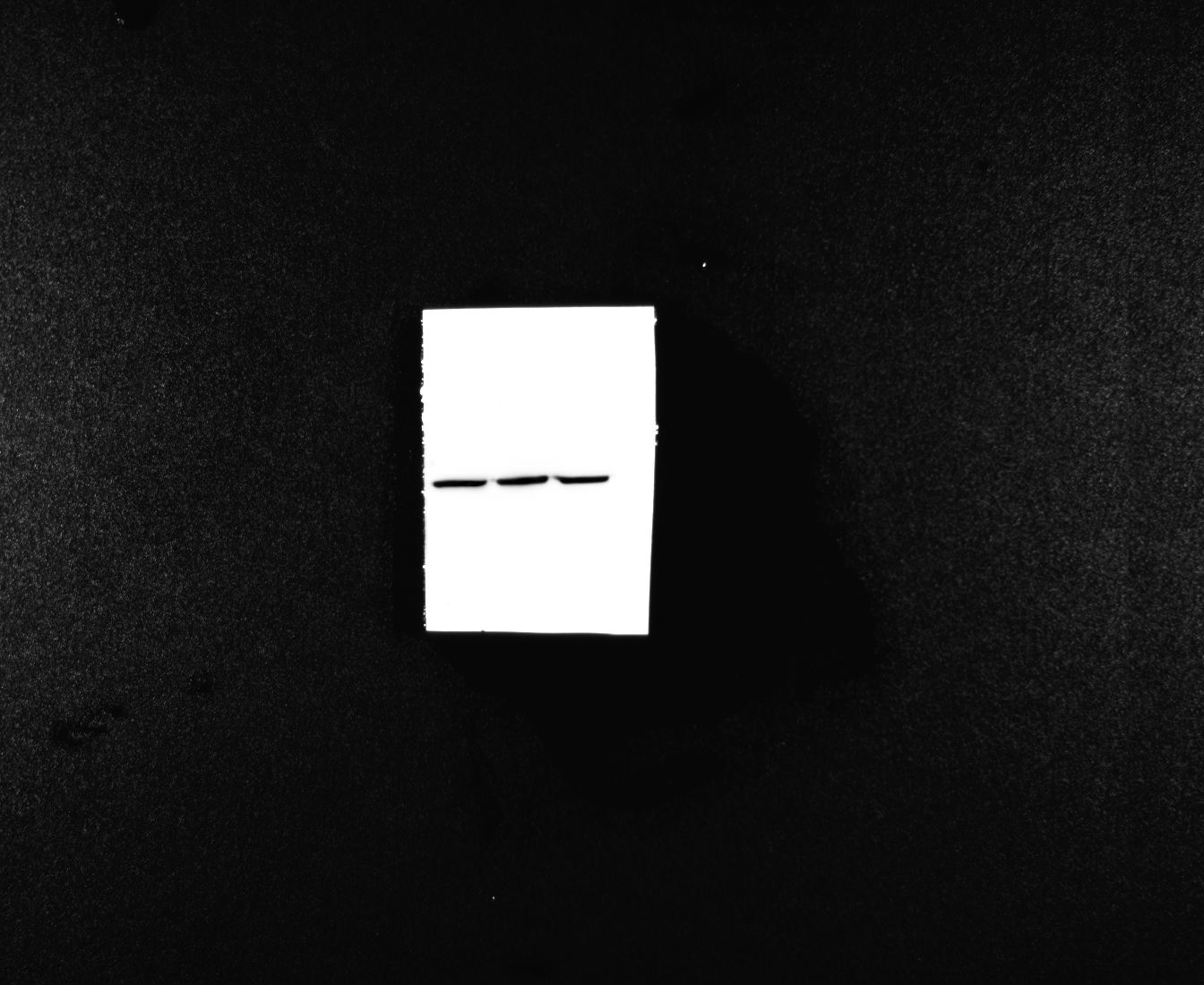


37 kD

GAPDH

Supplement: Supplementary Materials — Full scan of uncropped western blot images. [file 1680634.f1.docx]

## Slide 1
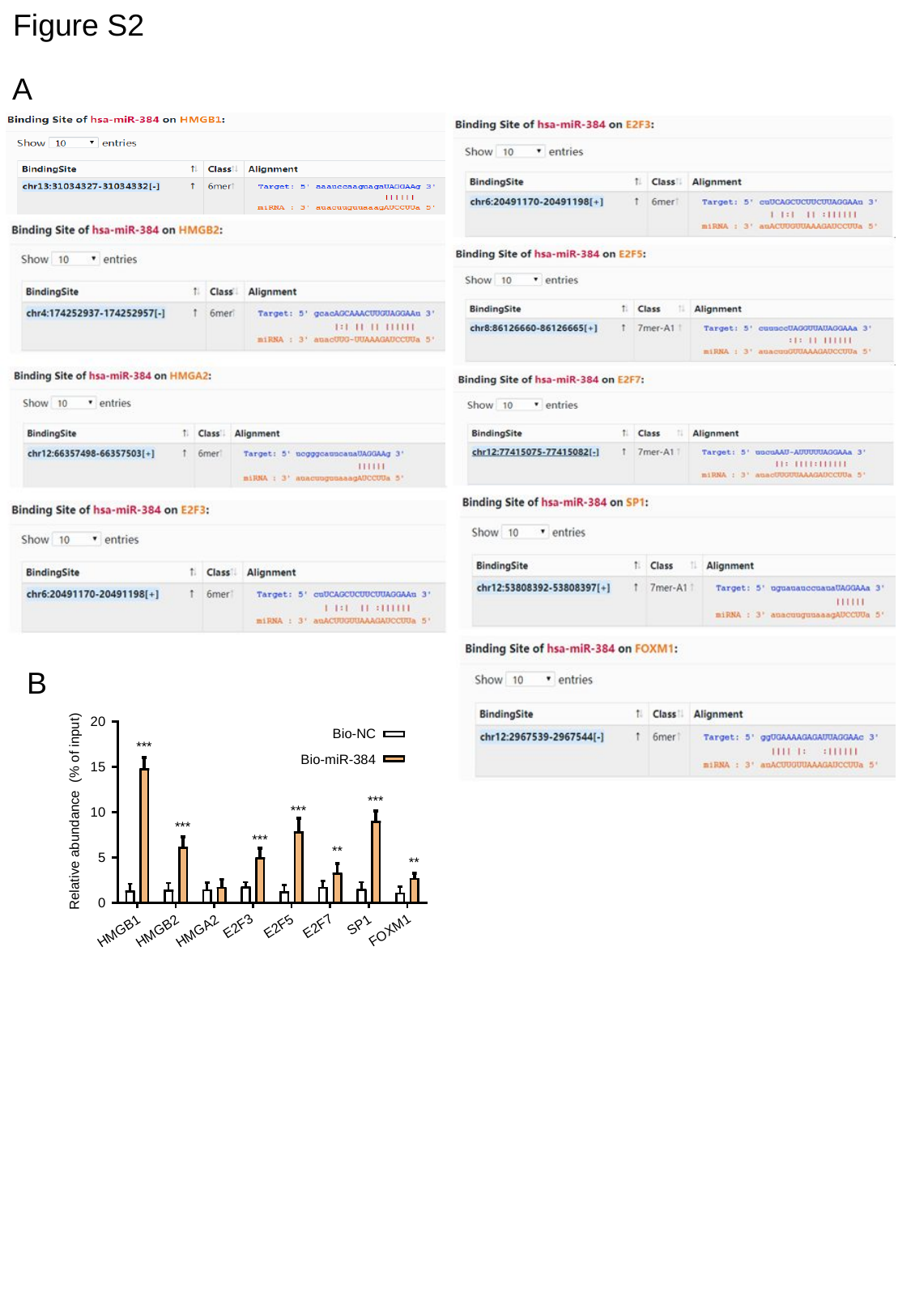

Figure S2
A
B
20
Bio-NC
***
Bio-miR-384
15
***
***
Relative abundance (% of input)
10
***
***
**
5
**
0
SP1
E2F3
E2F5
E2F7
FOXM1
HMGB1
HMGB2
HMGA2

Supplement: Supplementary Materials — Figure S1. Screening of potential interacting miRNAs of circ_0008285. A. Circinteractome database prediction results of the candidate binding miRNAs of circ_0008285. B. RNA-pull down analysis of the interacting miRNAs using biotin-circ_0008285 probe or control oligo in U251 cells. Data were normalized to the input sample. C. qRT-PCR analysis of circ_0008285 expression level after miR-384 overexpression. ∗∗stands for p < 0.01, ∗∗∗stands for p < 0.001. [file 1680634.f2.pptx]
