# Supplementary material for: circ_0008285 Regulates Glioma Progression via the miR-384/HMGB1 Axis
Source: Int J Genomics. 2023 Aug 3;2023:1680634. doi: 10.1155/2023/1680634 (PMC10415084; doi:10.1155/2023/1680634)
Supplement: Supplementary Materials — Figure S2. Screening of potential target mRNAs of miR-384. A. StarBase prediction results of miR-384 target mRNAs. B. RNA-pull down analysis of the interacting mRNA targets using biotin-miR-384 probe or control oligo in U251 cells. Data were normalized to the input sample. ∗∗stands for p < 0.01, ∗∗∗stands for p < 0.001. [file 1680634.f3.pptx]

## Slide 1
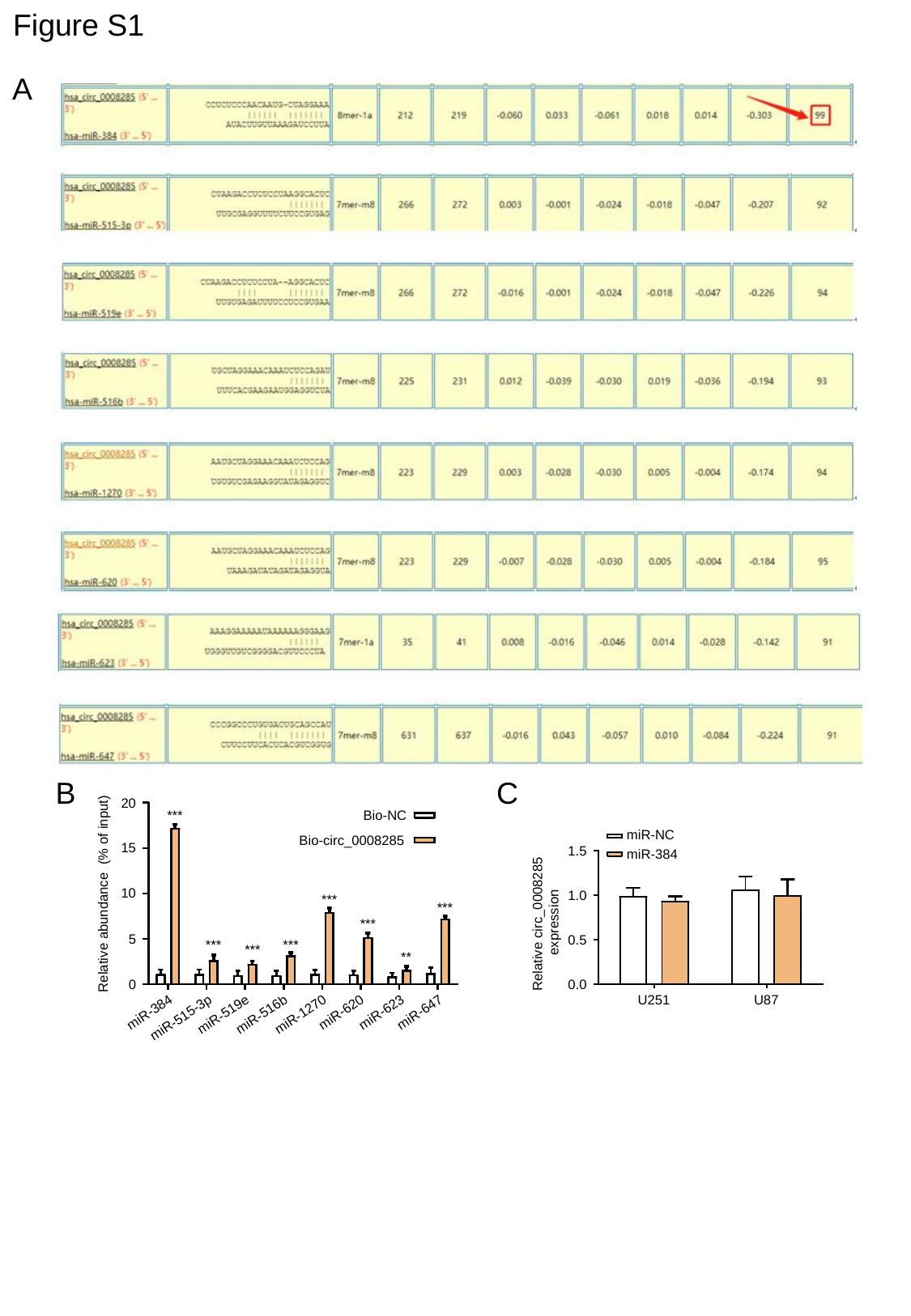

Figure S1
A
B
C
20
***
Bio-NC
miR-NC
1.5
miR-384
Relative circ_0008285
expression
1.0
0.5
0.0
U251
U87
Bio-circ_0008285
15
10
Relative abundance (% of input)
***
***
***
5
***
***
***
**
0
miR-384
miR-620
miR-623
miR-647
miR-519e
miR-516b
miR-1270
miR-515-3p
